# Establishing radiation therapy advanced practice in New Zealand

**DOI:** 10.1002/jmrs.33

**Published:** 2014-01-28

**Authors:** Karen Coleman, Marieke Jasperse, Patries Herst, Jill Yielder

**Affiliations:** 1 Department of Radiation Therapy, University of OtagoWellington, New Zealand; 2 University of AucklandAuckland, New Zealand

**Keywords:** Advanced practice, postgraduate qualification, profile criteria, radiation therapist

## Abstract

**Introduction:** Advanced practice (AP) is of increasing interest to many radiation therapists (RTs) both nationally and internationally. In New Zealand, initial research (2005–2008) showed strong support for the development of an AP role for medical radiation technologists (MRTs). Here, we report on a nationwide survey in which RTs validated and prioritised nine AP profiles for future development.

**Methods:** All registered RTs in New Zealand (*n* = 260) were invited to take part in a survey in December 2011; 73 of whom returned a complete response.

**Results:** RTs supported the implementation of AP roles in New Zealand and the requirement of a Master's degree qualification to underpin clinical knowledge. Most RTs endorsed the criteria attributed to each of the nine proposed AP profiles. The study identified that activities may qualify as either advanced practice or standard practice depending on the department. All participants agreed that an advanced practitioner should be a leader in the field, able to initiate and facilitate future developments within as well as outside this specific role. Acceptance of the AP roles by RTs and other health professionals as well as the availability of resources for successful implementation, were concerns expressed by some RTs.

**Conclusion:** The authors recommend (1) the development of one scope of practice titled ‘advanced practitioner’ with generic and specialist criteria for each profile as the future career pathway, (2) promotion and support for the AP pathway by the New Zealand Institute of Medical Radiation Technology and the New Zealand Medical Radiation Technologists Board.

## Introduction

The concept of advanced practice (AP) has gained momentum in several countries including the United Kingdom (UK), Canada and Australia in recent years. AP commenced in the UK where, due to health workforce issues in the last two decades, a four-tier career structure was introduced for radiographers to provide a multilevel model of service delivery.^1^ The aim was to provide an additional workforce to deliver the service and offer rewarding careers and lifelong learning for all practitioners.^1^,^2^ The undergraduate education and training of radiation therapists (RTs) differs between the UK and New Zealand (NZ), so skill levels do not always translate. Therefore, it is important that the RT profession identifies the clinical and education needs for AP based on their own population and health economy.^3^ This would take into account service requirements and needs for RT advanced practice, as well as the views of radiation oncologists, oncology nurses, medical physicists and other core professionals groups.

Previous research in New Zealand from 2005 to 2008 had led to a report to the New Zealand Institute of Medical Radiation Technology (NZIMRT) on role development and AP for both medical imaging and radiation therapy.^4^ The report confirmed findings from the UK that medical radiation technologists (MRTs) were capable of extending their roles into non-traditional areas and performing to high levels of expertise when they have undertaken appropriate postgraduate education and experience. It also highlighted the perception that New Zealand MRTs wish to obtain clinical advancement through a structured framework and that this would increase job satisfaction, recruitment and retention for the profession.^4^

In 2008, in collaboration with the NZIMRT, the University of Otago conducted a nationwide survey to canvas the opinion of RTs and radiation oncologists on role extension for RTs in NZ. The results indicated that the majority of RTs and radiation oncologists supported role extension and the development of advanced practitioner roles.^5^ This research confirmed the opinion that RTs were capable of taking on extended roles with appropriate education, training and support. Furthermore, there was strong support for the development of formalised postgraduate qualifications to underpin the clinical knowledge of an advanced practitioner.

The current research, again conducted in collaboration with the NZIMRT, builds on the previous advanced practice studies.^4^,^5^ RT working groups consisting of RTs interested in AP from three different departments, proposed nine RT advanced practice profiles and criteria for these AP roles. A nationwide survey was conducted to validate and prioritise these profiles for future development. All qualified RTs in NZ were invited to participate. The study further investigated the advantages and barriers that may affect the implementation of these profiles. This article reports on the results of the radiation therapy aspects of this research. A separate companion article will report the results for the medical imaging survey.^6^

## Method

Following ethical approval, granted by the Multi-region Ethics Committee (MEC/11/EXP/097) and Maori consultation with the Ngai Tahu Research Consultation Committee, an electronic questionnaire hosted on Survey Monkey-™ was distributed nationwide to all 260 registered RTs practising in NZ in December 2011.

The first section of the questionnaire contained questions addressing demographic and work factors, such as age, gender, department and work experience.

The second section of the questionnaire detailed criteria for nine suggested AP profiles: palliative, paediatric, brachytherapy, head and neck, breast, prostate, imaging and volumising, patient education and research. Each profile included criteria such as taking a lead RT role in planning and delivering treatment, advanced knowledge of anatomy, disease pathways and treatment options, liaison with a multidisciplinary team, advanced knowledge of acute and late side effects and their management, psycho-oncology, multicultural perspectives and knowledge of evidence-based practice and research. Participants were asked to indicate their agreement with the criteria and make suggestions for inclusion and removal. Participants were then asked to rate which profiles should be prioritised for the profession, on a scale from 1 to 9, with the option to rate profiles equally.

The third section of the questionnaire asked participants to identify the perceived advantages and potential barriers to implementing AP in NZ.

Incomplete responses were excluded and 73 complete responses (a response rate of 28%) were exported and analysed using SPSS 19 (Armonk, NY). A descriptive analysis was conducted to provide an overview of the participants' characteristics and endorsement of each of the profiles. An analysis of variance was conducted to establish the ranking of the profiles, whilst the qualitative responses were subjected to an analysis of content by an independent researcher.

## Results

### Participant demographics

The participants of this study predominantly identified themselves as New Zealand European; female, aged 20–49 (see Table 1). The majority of the participants were from public departments (87.7%) and identified as staff RTs (75.3%). The participants' range of work experience varied considerably with approximately 25% of participants having previous experience working in an extended role. The majority of this role extension had taken place in New Zealand (66.7%), and involved aspects of pre-treatment (50.0%).

**Table 1:** Participant demographics

	*N* = 73	%
Gender, female	61	84
Ethnicity, New Zealand European	62	85
Age (years)		
20–29	25	34
30–39	23	32
40–49	15	21
50–59	6	8
≥60	3	4
Work experience (years)		
1–5	20	27
6–10	16	22
11–15	16	22
16–20	6	8
>20	15	21
Public departments	64	88
Position		
Staff RT	55	75
Experience with role extension	18	25
Role extension in pre-treatment	9	50
Role extension in New Zealand	12	67*

* Percentage of 18 participants with role extension experience.

### Endorsement of the AP profiles and criteria

For the nine profiles, there were generic and specialised criteria for participants to confirm or comment on. The generic criteria were the following:

- Lead RT in the particular specialisation
- Advanced knowledge of the particular specialisation
- Liaison with the multidisciplinary team
- Knowledge of patient journey, psycho-oncology and survivorship
- Knowledge of evidence-based practice, research and ongoing education
- Knowledge of multicultural perspectives

Additional criteria relating to the specialisation were proposed for each profile.

For each of the profiles more than half the number of participants (59–84%) endorsed the criteria and had no suggestions for addition or removal (see Table 2). In the open-ended option at the end of each profile, a small number of participants (<5%) specifically mentioned that some of the suggested criteria within the proposed AP profiles were already part of standard RT practice in their department. For example:

**Table 2:** Endorsement of the advanced practice profiles

Proposed profile	Endorsement (%)
Research	84
Brachytherapy	77
Head and neck	71
Prostate	70
Palliative	67
Breast	67
Patient education	67
Imaging and volumising	66
Paediatric	59

*A great deal of these are not advanced practice but are included in a current RTs role* (RT 26).

Concern was also expressed that the implementation of AP positions may reflect poorly on standard RTs:

*All of the above aspects should be an integral part of all radiation therapists role. By removing these and making them ‘advanced practice’, this will dumb down the profession for anyone who is … JUST a radiation therapist* (RT 56).

The importance of clarity was also emphasised: *There is much talk of advanced practice and yet it does not seem to be clearly defined* (RT 68). Participants were also asked to identify if there were any potential AP profiles missing. Suggested profiles were stated in the areas of information technology (IT) and new technology education (8.2%), site specialist (4.1% gynaecology) and quality assurance (4.1%).

### Potential prioritisation of AP profiles

An analysis of variance revealed no significant difference between the prioritisation of each of the proposed profiles, *F*(8, 643)=1.59, *P*=0.13. This suggests that all the profiles were endorsed to a similar extent.

### Perceived advantages of implementing AP in NZ

The perceived advantages of implementing AP in NZ included professional development and career progression, job satisfaction, retention, recognition and respect, in addition to increased departmental efficiency, quality of patient care and international standing and recognition (see Table 3).

**Table 3:** Responses to implementing advanced practice in New Zealand

	*N* = 73	%*
Advantages of implementing advanced practice		
Professional development and career progression	30	41
Job satisfaction	24	33
Enhanced patient care	23	32
Departmental efficiency	23	32
Retention	8	11
Recognition and respect	5	7
International standing and recognition	5	7
Barriers to implementing advanced practice		
Resources and remuneration	37	51
Acceptance from other disciplines	26	36
Resistance of some RTs	14	19
Culture of the department	13	18

* Unprompted responses only.

The most prevalent perceived advantage of implementing AP in NZ was the opportunity to develop professionally and address the seeming lack of career progression and respect within the profession. For example:

*Role development and growth. I feel that once you become a RT and have a few years' experience there isn't much more to advance to, except to a grade position which there are limited numbers of* (RT 22).

*The profession becomes less of a glorified button pusher* (RT 72).

The opportunities for career progression were also perceived to have significant implications for job satisfaction and retention within the profession:

*Main advantage would be greater career progression and satisfaction. RTs will leave the profession or move overseas if they cannot advance their careers here* (RT 15).

Another significant advantage of implementing AP identified by participants was the implications for increased departmental efficiency and enhanced patient care:

*Better flow of patient care as the person in the role will understand all aspects of the patient's journey. I think this understanding will also lead to better department efficiency* (RT 4).

There was also a desire for international standing and recognition, with one RT commenting that: *It will lead to a more highly educated workforce with greater skill and knowledge. Will allow NZ to compare with the rest of the world* (RT 9).

### Perceived barriers to implementing AP in NZ

The perceived barriers to implementing AP in NZ concerned the availability of resources and remuneration, in addition to aspects of the environment such as the ‘culture’ of departments, resistance from within the profession, and acceptance from other disciplines (see Table 3).

Many participants expressed their concern with respect to the resources involved in the training, implementation and remuneration of AP candidates, saying for example that:

*Budgets, size of departments, staff turnover, poor training or irrelevant training, time to complete qualification, cost of the qualification, education resources at local hospitals, support in the work place* (RT 3).

A significant number of participants also discussed the ‘culture’ within departments, resistance from within the profession, and acceptance from other disciplines, commenting on factors such as lack of support and resistance to change:

*Lack of support from the department as a whole – the culture of the department needs to be in support of these specialty roles and this comes from believing in their importance* (RT 4).

*The inevitable resistance to change, that will be found among, doctors, nurses, medical physicists and even some radiation therapists* (RT 53).

## Discussion

### Profiles and criteria

Overall there was strong support for the implementation of RT advanced practice in New Zealand. More than half the participants agreed with the criteria attributed to each of the nine proposed profiles. Results indicated that AP would be department-dependent with activities qualifying as advanced practice in some departments being considered standard practice in others. An example of this was the area of brachytherapy, as not all New Zealand radiation oncology departments specialise in this type of radiation therapy. Similarly, what may be an advanced technical skill today may be standard practice in the near future. For example, 3D radiation therapy planning is a skill which was seen as advanced practice in 2000, but is now part of the undergraduate programme and is standard practice.^2^

A few participants thought some criteria were already standard practice and there was concern that having an AP role would devalue the skills of the staff RT. It seems that there is confusion with much of the terminology; where the term ‘advanced practice’ is being used to describe what is essentially role extension.

On the basis of the 2008 report^4^ on role development and a possible career structure for MRTs, the NZIMRT approved the generic elements of an AP role as:

- Clinical leadership
- Teaching and supervision
- Legal and ethical issues
- Quality assurance
- Ongoing supervision and moderation
- Clinical decision-making
- Professional and current issues
- Research and evidence-based practice
- Clinical skills and theory to support
- Master qualification

This template was based on recommendations from work completed in the UK^7^ and is in line with Canada^8^ and Australia^2^ on the implementation of advanced practice. Internationally an advanced practitioner is deemed to be a leader in the field, able to initiate and facilitate future developments within as well as outside their specific role.^4^ A key aspect of the AP role is ‘clinical leadership’, which means keeping up to date with the latest research, interacting at a high level with professionals, educating others and contributing to developments in practice,^2^ as opposed to a focus on specific clinical skills that may change with time. Therefore, the concept of one scope of AP with key generic criteria would be the basis of an advanced practitioner role, with specialist criteria for each accepted profile. The AP scope of practice would be facilitated by the NZ Medical Radiation Technologists Board (MRTB).

There were several other profiles suggested, mostly in the areas of IT and new technology, site specialist and quality assurance. However, with the AP role focusing on generic elements, expertise can be translated into other specialist areas of radiation therapy. Where knowledge and clinical expertise is in one particular area, this has been defined as role extension and it may give rise to the profession considering a title to acknowledge this, for example ‘clinical specialist’.

In New Zealand, there are areas of role extension that have developed, due to staffing and department efficiency. An example is the RT-led treatment review clinics in a public hospital that does not have radiation oncology registrars.^9^ RTs have an extensive ‘in-house’ training to underpin and expand existing knowledge, so that they are confident and competent to accurately and effectively interpret information reported by patients during the treatment review clinic.

### Advantages

This current research project has demonstrated that NZ RTs are aware of AP and the advantages this would bring to the profession. Job development and career structure, job satisfaction, enhanced patient care and department efficiency were highlighted by the participants as advantages to having AP roles. These responses were unprompted and came from an open-ended question summarised in Table 3. The findings support the advantages for AP indicated by New Zealand MRTs in 2008^4^ and also in the 2009 nationwide survey of RTs and radiation oncologists.^5^

The skill-mix model from the UK has been viewed widely as a catalyst for AP in many countries.^1^ Most UK developments occurred due to the growth in imaging services, shortage of Radiologists, the need for radiographer career progression and the desire to have an efficient patient pathway.^1^,^3^ Similarly, in Canada advanced practice RT roles have been implemented to free up radiation oncologists' time. In this way, radiation oncologists can pursue more complicated cases, see more new patients, which in turn will reduce wait times for services and increase access for patients.^10^

In contrast, NZ RTs did not identify retention, recognition, respect and international standing as important advantages of AP.^11^ Similarly, the interest into AP roles was not driven primarily by freeing up the time of radiation oncologists. This may be due to the interpretation of what constitutes AP being defined differently in different countries. For example, RT planning is a key component of the undergraduate RT curriculum in New Zealand, whereas in the UK it is often an area led by physicists. However, it is noted that this is changing in the UK with planning now a part of the Health and Care Professions Council standards of proficiency.^12^

### Barriers

In the current research there were perceived barriers that were highlighted strongly by the RT participants. Resources and remuneration were reported by many as a barrier to implementing AP roles. This was an interesting finding as in the parallel medical imaging survey;^6^ resources and remuneration were reported as a barrier by considerably fewer of the respondents. It could be interpreted that as there are only eight radiation oncology departments in NZ, a relatively small workforce and at times a high turnover rate, could create concern about the employers' commitment to support AP roles in the longer term. In the current economic environment, the implementation of AP roles may be seen as setting up a system that would be more costly. However, the establishment of AP roles may well improve workflow, create efficiencies and enhance patient care, as shown in a recent Canadian study.^10^

Some RTs expressed concern that radiation oncologists, nurses and even some of their own colleagues would resist RT role extension and AP roles. Interestingly, our earlier research^5^ indicated that radiation oncologists supported RT role extension and AP roles to a greater extent than the RTs themselves. Resistance from RTs was identified as the most significant barrier^5^ whereas less than a quarter of RTs in this current study thought that there would be resistance from within the profession.

The culture and size of the department was also perceived as a barrier. Being able to release RTs for further training and education and having a supportive structure in place for the AP role would be ways in which a department could support the implementation of advanced practice.^11^

### Postgraduate qualifications

The minimum qualification for AP roles has been accepted by the NZIMRT as a Masters qualification. Professional organisations in the UK and Australia^2^ also recommend that a Masters degree is the academic qualification that is required for advanced practitioners. However, for most RTs undertaking extended roles currently, the formal postgraduate qualification is recommended but not required. Many RTs working in extended roles clinically are completing postgraduate academic papers from universities to obtain advanced knowledge in particular areas.^13^ Therefore, the more common pattern is for a clinical department to develop a role for advanced skills, based on the needs of the department. Examples are palliative and site-specific RTs. In Canada, similar roles have been developed with pilot funding from provincial government and have been titled Clinical Specialist RTs,^14^ with radiation oncologists providing ‘on the job’ training and supervision.

**Figure 1:**
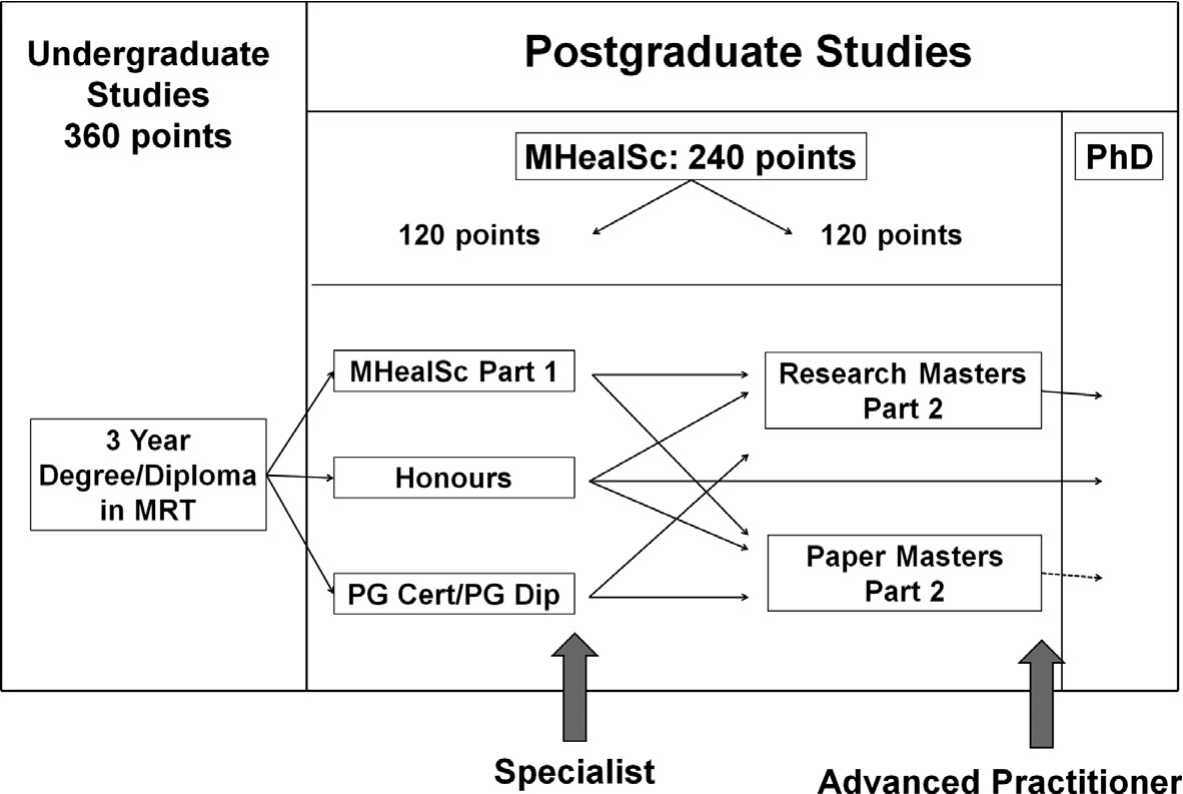
Postgraduate pathways – University of Otago.

It may be that in the NZ context, a specialist role is created at postgraduate diploma level for those RTs with role extension skills, while the overall leadership role of the AP requires the Master's degree (see Fig. 1). The specialist role would give recognition to those who are deemed experts in a particular skill area.

The University of Otago has established postgraduate papers and pathways in RT advanced practice that would lead to a Masters qualification. The two pathways are namely:

- Honours (primary research by thesis)
- RT advanced practice papers

These pathways lead into a research or paper-based Masters. By providing flexibility and options, the postgraduate programme can be adapted to the relatively small numbers of RTs in NZ interested in seeking academic qualifications which can be used towards the advanced practitioner role.

### Limitations

One limitation of this study concerned the way in which participants were asked to rate which profiles should be prioritised for the profession. The question consisted of a scale from 1 to 9, but the option to rate profiles equally was presented. Giving the option to rate profiles equally is likely to have contributed to the lack of prioritisation of profiles for the profession.

## Conclusion

Nine proposed advance practice profiles were validated by qualified RTs in New Zealand with additional profiles identified in the areas of IT and new technology, site specialist and quality assurance. This research supports the implementation of AP roles in NZ with a Master's degree as the minimum qualification to underpin clinical experience. We would like to promote one scope of practice, titled ‘Advanced Practitioner’, with generic profile criteria as its basis and specialist criteria for each accepted profile. This AP scope of practice would be facilitated by the Medical Radiation Technologists Board with a process to ensure appropriate standard of practice and specific Continuing Professional Development for an AP role.

RT advanced practice supports the desire for RTs in NZ to increase their career opportunities. However, this will need support from colleagues, management and government to facilitate training, education and structuring of roles long term.

Evidence from this research shows that there is interest among RTs for an AP scope of practice in New Zealand. A flexible academic pathway for RT postgraduate qualifications has been developed. Therefore, it is imperative for the profession and stakeholders to champion the scope of practice for AP; so that RTs can fully develop in these areas.

### Recommendations

There are several recommendations that emerge from this research. These have been crafted jointly with the companion medical imaging article,^6^ as it is important that career development is considered as an integrated and consistent model for the whole profession. The authors recommend that:

1. The NZIMRT and MRTB promote and support the development of an AP pathway for radiation therapy in New Zealand.
2. There is one advanced scope of practice, titled Advanced Practitioner for the future career pathway, with generic and specialised criteria for each accepted profile.
3. A Master's degree is the educational requirement for an AP role.
4. A postgraduate diploma is the educational requirement for Specialist roles; for practitioners undertaking extended role activities but not in a formalised AP position.
5. The MRTB develop appropriate standards of practice and specific continuing professional development requirements for the AP role.
6. The University of Otago works with clinical radiation therapy departments to identify service needs for AP roles.
7. Funding is identified to support the education and training required for each AP role.

## Funding Information

This study was partially funded by the NZIMRT.
